# Comparative transcriptional profiling of orange fruit in response to the biocontrol yeast *Kloeckera apiculata* and its active compounds

**DOI:** 10.1186/s12864-015-2333-3

**Published:** 2016-01-04

**Authors:** Pu Liu, Kai Chen, Guofeng Li, Xiaoping Yang, Chao-an Long

**Affiliations:** Key Laboratory of Pomology, Anhui Agricultural University, Hefei, 230036 P. R. China; Key Laboratory of Horticultural Plant Biology of the Ministry of Education, National Centre of Citrus Breeding, Huazhong Agricultural University, Wuhan, 430070 P. R. China; Research Institute of Fruit and Tea, Hubei Academy of Agricultural Science, Wuhan, 430064 P. R. China

**Keywords:** Biological control, *Kloeckera apiculata*, Plant defence, Postharvest, Citrus

## Abstract

**Background:**

The yeast *Kloeckera apiculata* strain 34–9 is an antagonist that shows biological control activity against the postharvest fungal pathogens of citrus. An antifungal compound, 2-phenylethanol (PEA), has been identified from the extract of *K. apiculata*. To better understand the molecular processes underlying the response of citrus fruit tissue to *K. apiculata*, the extract and PEA, microarray analyses were performed on navel oranges using an Affymetrix Citrus GeneChip.

**Results:**

As many as 801, 339 and 608 differentially expressed genes (DEGs) were identified after the application of *K. apiculata,* the extract and PEA, respectively. In general, *K. apiculata* induced the expression of defence-related genes. In addition to chitinase and β-1,3-glucanase, genes involved in ethylene (ET), jasmonic acid (JA), calcium signalling, MAPK signalling and phenylalanine metabolism were induced. In contrast, monodehydroascorbate reductase, superoxide dismutase (SOD), catalase (CAT), peroxidase (POD) and carotenoid biosynthesis genes were down-regulated. The expression profiles for the extract- and PEA-treated samples were similar to that found for yeast (sharing 57.4 % DEGs), with a significant increase in the transcript levels of defence-related genes.

**Conclusion:**

This study provides a global picture of the gene expression changes in navel oranges after the application of the antagonist yeast *K. apiculata*, its extract and PEA. The interpretation of the DEGs revealed new insight into the molecular processes that regulate the defence responses in orange tissue.

**Electronic supplementary material:**

The online version of this article (doi:10.1186/s12864-015-2333-3) contains supplementary material, which is available to authorized users.

## Background

The biological control of postharvest pathogenic fungi using microbial antagonists has recently emerged as a promising alternative to the use of synthetic fungicides [1–4]. Over 30 yeasts have been isolated and investigated for their biological control efficacy against postharvest fruit diseases. *Kloeckera apiculata* strain 34–9, a yeast isolated from the epiphytes of citrus roots [5], has been shown to suppress postharvest fungal pathogens in citrus, e.g., *Penicillium digitatum* and *Penicillium italicum*, the causal agent of green and blue mold, respectively [6, 7].

Knowledge regarding the modes of action of biological control agents is essential for developing appropriate commercial formulations and application methods to maximize the potential use of biological control. Several routes have been proposed to explain the action mechanism of biological control agents. The yeast-induced defence response of fruit has been considered a potential means to suppress infection with plant pathogens, and growing evidences have supported this point of view [1–4]. *Pichia guilliermondii* and *Candida famata* enhanced the accumulation of phytoalexins, scoparone and scopoletin in citrus wound tissues [8, 9]. Nantawanit et al. [10] reported that *P. guilliermondii* induced a defence response in chili fruit against *Colletotrichum capsici*. The yeast *Aureobasidium pullulans* can induce the accumulation of chitinase, β-1,3-glucanase and peroxidase in apple fruit [11]. *Candida saitoana* induced defence responses in apple fruit [12]. *Rhodosporidium paludigenum* induced resistance and defence-related responses against *P. digitatum* in citrus fruit [13]. The biocontrol capability of *Pichia caribbica* was based on the activation of defence-related enzymes in peaches [14].

Reactive oxygen species (ROS) and the phytohormones signalling pathway have been shown to regulate the yeast response processes [15]. Castoria et al. [16] indicated that the ability to tolerate high levels of ROS production in fruit tissue is an essential characteristic of effective yeast antagonists. Macarisin et al. [17] reported that yeasts on the surfaces of fruit produced H_2_O_2_ and O_2_^−^; O_2_^−^ acted as a global regulator to activate the fruit defence responses. The application of *P. membranefaciens* to citrus fruit enhanced the levels of H_2_O_2_ and O_2_^−^ in the host tissue [18]. In contrast, fungal pathogens suppress the host tissue defence responses by acidifying the fruit with organic acids, such as citric and gluconic acid [19, 20]. The acidification of the tissue might suppress host cells’ production of H_2_O_2_, and enhance the sensitivity to pathogen-produced pectolytic enzymes [21–23].

Three phytohormones, salicylic acid (SA), jasmonic acid (JA) with its derivatives (collectively called jasmonates) and ethylene (ET), have been shown to play major roles in regulating defence responses in plants [24–26]. *Candida oleophila* induced disease resistance by increasing the production of phytoalexin, ET biosynthesis and phenylalanine ammonia-lyase (PAL) activity [27]. Preharvest treatment with SA and methyl jasmonate (MeJA) induced defence-related enzymes in sweet cherries [28]. The integration of antagonistic yeast with SA or JA resulted in a remarkably improved biocontrol efficacy [29, 30]. In general, the SA-signalling pathway is believed to mediate the resistance to biotrophic pathogens, whereas the JA/ET-signalling pathway is thought to be necessary for resistance to necrotrophic pathogens [26]. This regulation consists of the control of a complex regulatory network that connects the different pathways to enable each to assist or antagonize the others as required and fine-tune the defence response to the individual pathogen. Other plant hormones, including abscisic acid (ABA), gibberellin and auxin, act as moderators of the plant immune signalling network and have also been implicated in plant defence [31].

Many classes of compounds derived from *Trichoderma* strains, including proteins, peptaibols, oligosaccharides, low-molecular-weight compounds and small secondary metabolites, can elicit plant defence responses [32], such as the expression of pathogenesis-related (PR) proteins, the induction of lignification and ROS-accumulation. Several studies have reported global changes in fruit gene expression in response to adverse stress [33–37] and antagonist yeasts [38–41] by using proteomic and transcriptomic analyses. Many fruit defence response genes and proteins were identified that may increase fruit resistance; however, little is known regarding the molecular basis of functional compounds from antagonist yeast underlying the induction of host responses. An antifungal compound 2-phenylethanol (PEA) was previously identified from the extract of *K. apiculata* [42]*.* The present study was undertaken to provide a systematic view of the citrus response to the yeast *K. apiculata* and its functional compounds by using an Affymetrix Citrus GeneChip.

## Results

### Global changes in citrus gene expression profiles

**Fig. 1:**
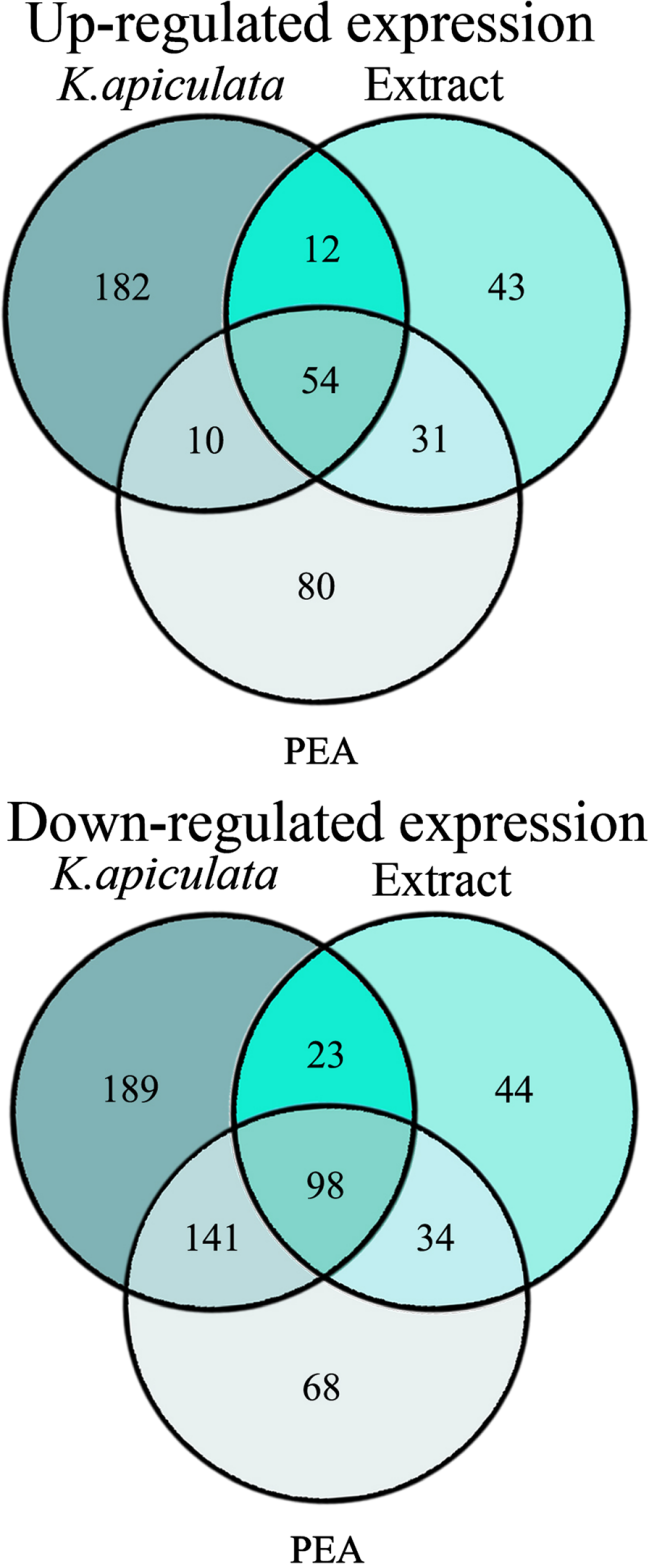
Number of differentially expressed genes (DEGs) in citrus after statistical analysis. Venn diagram shows the number of up-regulated and down-regulated genes that are expressed in common or in special between *K. apiculata*, 2-phenylethanol (PEA) and the extract treatment

To obtain an overall picture of the gene regulation, biocontrol yeast, the ether extract and active compound were used to treat citrus and two independent microarray analyses were performed for each treatment. To reduce experimental variation, two sets of six fruit exocarps were harvested from each treated and untreated (water control) fruit. After the removal of low-quality and internal reference probes, a total of 20,083 transcripts were reliably detected in the microarray analysis. Microarray analysis gene changes in citrus exocarp revealed as many as 801, 339 and 608 differentially expressed genes (DEGs) that showed a significant (*P* < 0.05) change in expression (≥1.3-fold) after 24 h of incubation with *K. apiculata,* the extract and PEA (Fig. 1). We further analysed these genes in subsequent experiments.

**Fig. 2:**
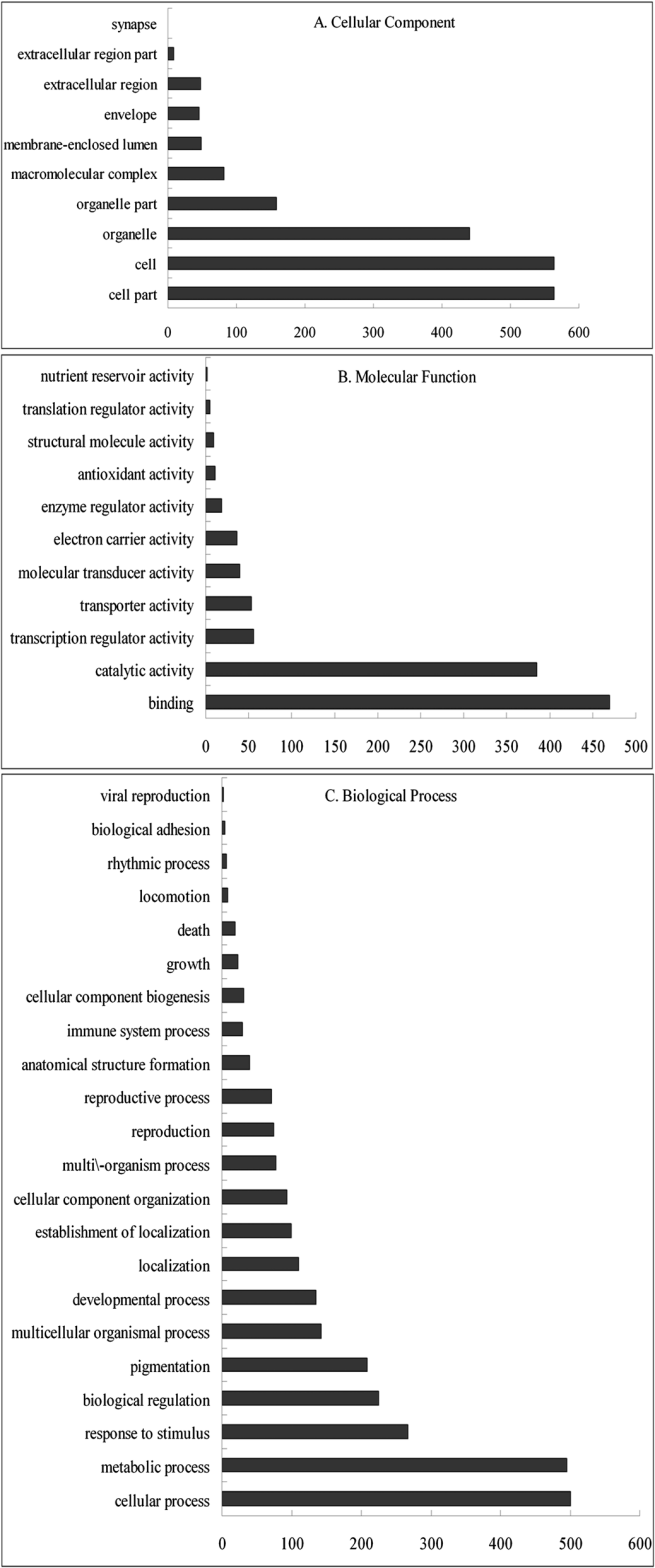
Functional categorization of global pattern of gene expression in citrus in response to different treatment based on GO annotation

All DGEs were aligned against the *Arabidopsis* database by using the Citrus HarvEST software, and detailed descriptions of the sequences are shown in Additional file 1. GO categories were assigned to the 1052 DEGs using the Blast2GO program (http://www.blast2go.org). The DEGs were categorized into 22 groups based on their biological processes, as shown in Fig. 2. Response to stimulus (267), metabolic process (495), cellular process (501), pigmentation (208), biological regulation (225), multicellular organismal process (142) and developmental process (135) were the major categories. Categories based on the cellular component revealed that the responsive genes were mainly related to cell (564), cell part (564), organelle (440) and organelle part (159). With regard to molecular function, the DEGs were classified as catalytic activity (385), binding (470), transcription regulator activity (56), transporter activity (53), molecular transducer activity (40), electron carrier activity (36), enzyme regulator activity (19), antioxidant activity (11), structural molecule activity (9), translation regulator activity (5) and nutrient reservoir activity (2). Of these, the plastid and intracellular organelle were the major sub-cellular organelles involved in the citrus response.

The responsive genes were further assessed using KEGG pathway analysis (http://www.genome.jp/kegg/) (Additional file 1). The most represented pathways are phenylpropanoid biosynthesis (26), limonene and pinene degradation (18), ABC transporters (14), proteasome (3), lysosome (5), oxidative phosphorylation (9), flavonoid biosynthesis (20), the regulation of autophagy (3), calcium signalling pathway (13), apoptosis (18), fatty acid metabolism (7), MAPK signalling pathway (25), phenylalanine, tyrosine and tryptophan biosynthesis (7), citrate cycle (TCA cycle) (5), flavone and flavonol biosynthesis (14), starch and sucrose metabolism (13), arachidonic acid metabolism (4), phenylalanine metabolism (7), ascorbate and aldarate metabolism (6) and carotenoid biosynthesis (5). Most of these pathways were consistent with biological processes that were already identified by GO analysis. Some of these pathways were related to the defence response based on previous knowledge, such as phenylpropanoid biosynthesis and the calcium signalling pathway [26, 31, 32, 36].

### Change pattern of gene expression in citrus in response to *K. apiculata*

**Fig. 3:**
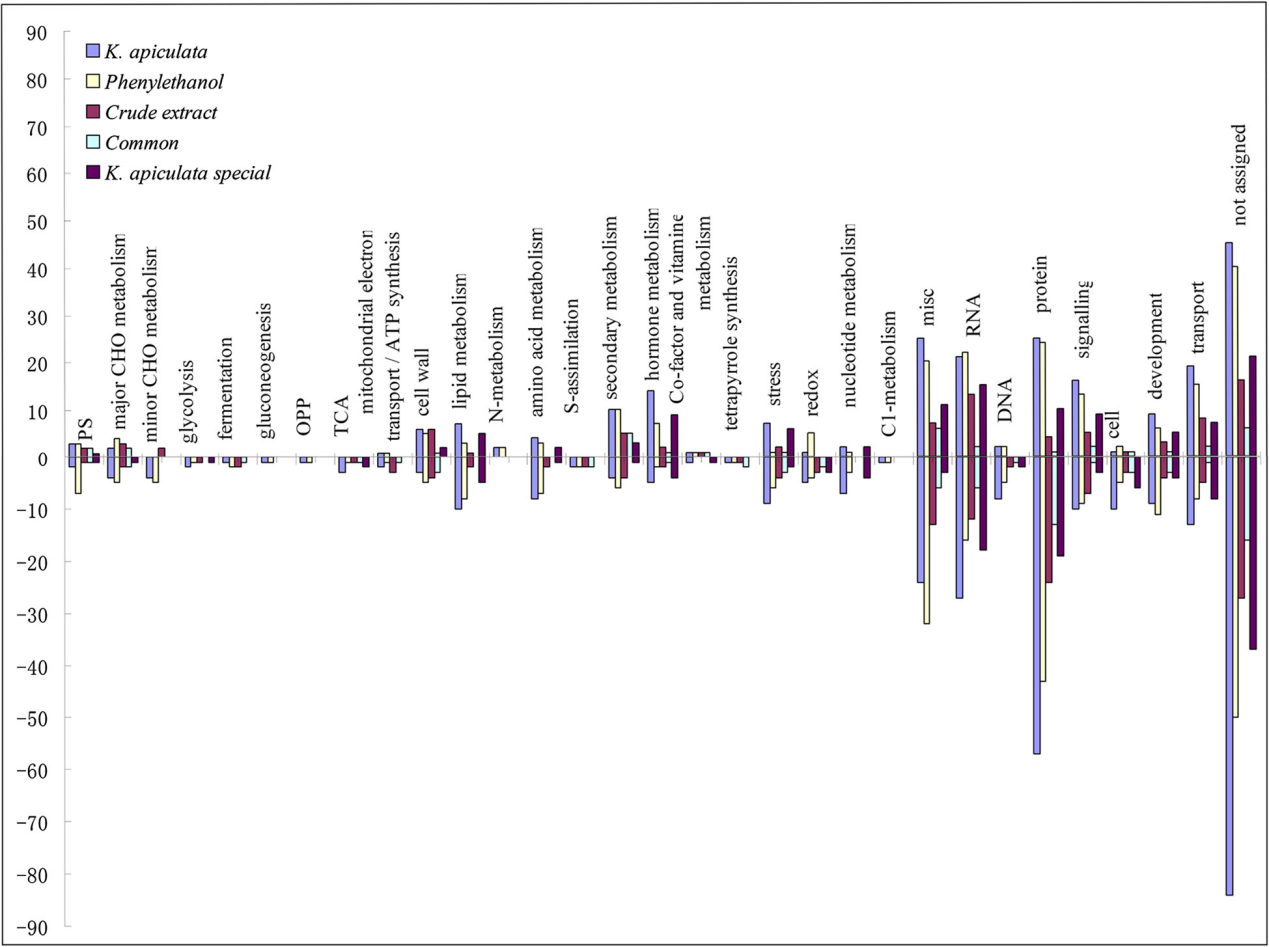
MapManBin analyses of the common or in special up-regulated and down-regulated genes in citrus between *K. apiculata*, PEA and the extract treatment

**Fig. 4:**
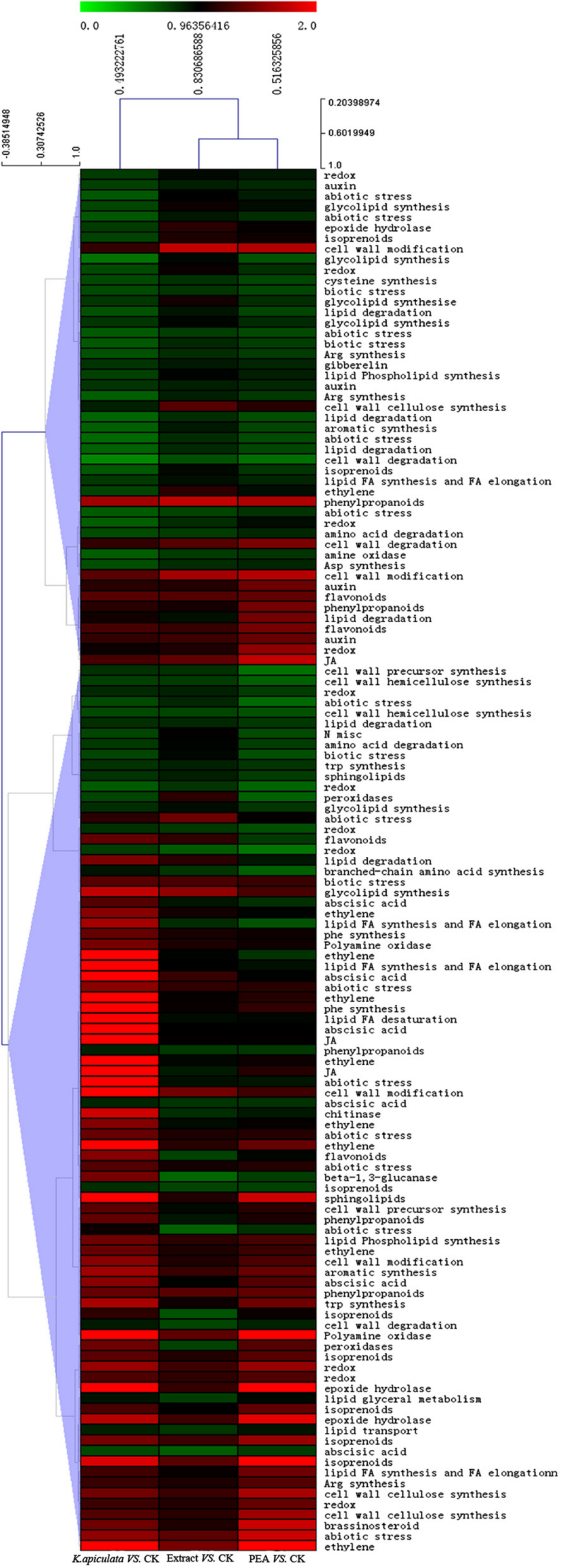
Cluster analysis of the expression profiles of resistance-related differentially expressed genes in citrus by MeV (http://www.tm4.org/mev.html). Each column represents a sample, and each row represents a single gene. The diagram was generated using log_2_-transformed ratio values, and colours indicate relative signal intensities. Genes down-regulated in the treatment compared to control are depicted in green, and up-regulated genes are depicted in red

Of the 801 DEGs in orange exocarp tissue treatment with *K. apiculata*, 56 % of the annotated genes were down-regulated and 44 % were up-regulated. Furthermore, the microarray data for the probes of the significant dataset were mapped to *Arabidopsis* using the MapManBin software (http://ppdb.tc.cornell.edu/dbsearch/searchacc.aspx). The data obtained from this analysis are presented in Fig. 3 and Additional file 2. Major and minor CHO metabolism (10), glycolysis (3), fermentation (1), TCA (3), mitochondrial electron transport/ATP synthesis (3), lipid metabolism (17), amino acid metabolism (12), redox (6), nucleotide metabolism (9), DNA (10) and proteins (82) associated with the cell (11) showed more down-regulated than up-regulated genes, while more up-regulated genes were found in PS (5), N-metabolism (2), hormone metabolism (17) and secondary metabolism (14) associated with the cell wall (9) in response to *K. apiculata* treatment. A number of new genes that are potentially related to defence responses were identified in this study. Based on microarray and previous data [26, 31, 32, 36], hormone, reactive oxygen species (ROS), lipid, secondary metabolite, cell wall, stress, phenyalanine metabolism related genes were selected for further analysis. Figure 4 and Additional file 3 summarize the changes in these defence-related genes.

The first noticeable pathway is the hormone metabolism pathway. In total, 17 differentially expressed genes are involved in hormone metabolism, including the ethylene (ET)*-*signalling pathway of eight *ethylene response factor (ERF)* genes (Cit.18086.1.S1_at, Cit.22763.1.S1_s_at, Cit.2675.1.S1_s_at, Cit.2677.1.S1_at, Cit.17142.1.S1_s_at, Cit.18673.1.S1_at, Cit.20640.1.S1_at, Cit.16845.1.S1_at); jasmonic acid (JA)-signalling pathway of one *hydroperoxide lyase* (*HPL*) (Cit.10444.1.S1_at) and five *allene oxide synthase (AOS)* genes (Cit.905.1.S1_at, Cit.6011.1.S1_at, Cit.23585.1.S1_at, Cit.31140.1.S1_at, Cit.996.1.S1_s_at) and five abscisic acid (ABA)-signalling pathway genes (Cit.13424.1.S1_at, Cit.5225.1.S1_at, Cit.10675.1.S1_at, Cit.13166.1.S1_at, Cit.34429.1.S1_s_at) (Fig. 4 and Additional file 3). Most of these genes were highly expressed in yeast-treated orange tissue, such as *ERF* (Cit.2677.1.S1_at), which was up-regulated 4.0-fold according to the microarray data. In addition, two polyamine (polyamine oxidase), three auxin-responsive and five gibberellic acid (GA; gibberellin receptor, gibberellin oxidase) genes were down-regulated.

Reactive oxygen species (ROS) accumulation has been well studied for biocontrol yeast-induced defence responses in fruits [15–18]. The second group of metabolic pathways is involved in the redox and antioxidation pathway. In total, five genes involved in antioxidant biosynthesis were down-regulated, including monodehydroascorbate reductase (Cit.3320.1.S1_s_at, Cit.3318.1.S1_at), superoxide dismutase (SOD, Cit.5267.1.S1_at), catalase (CAT, Cit.8351.1.S1_s_at) and peroxidase (POD, Cit.8515.1.S1_s_at) (Fig. 4 and Additional file 3). For example, SOD (Cit.5267.1.S1_at) was down-regulated 5.5-fold according to the microarray data, and the qRT-PCR data were consistent with these results, demonstrating that the level of SOD was 2.2-times lower in response to *K. apiculata*-treatment than in CK. Moreover, Cytochrome P450 plays an important role in the redox pathway and has been well characterized [43–45]. Over nine different cytochrome P450 genes were detected in our microarray data, such as monooxygenase/p-coumarate 3-hydroxylase, monooxygenase 83B1 and ent-kaurenoate oxidase.

The third group of metabolic pathways consists of signalling pathway and pathogenesis-related (PR) proteins. The signalling pathway genes included 13 genes for calcium and 25 genes for MAPK signalling, most of which were up-regulated in response to *K. apiculata* application. The other genes encoding for chitinase (Cit.15242.1.S1_at, 1.8-fold) and β-1,3-glucanase (Cit.10558.1.S1_s_at, 1.4-fold) were stimulated by *K. apiculata* application (Fig. 4 and Additional file 3). In addition, five different disease resistance protein genes (TIR-NBS-LRR class) were also found in our study.

The fourth group of significant *K. apiculata*-responsive genes included secondary metabolic processes, such as the phenylpropanoid pathway, limonene and pinene degradation, flavone and flavonol biosynthesis and carotenoid biosynthesis (Fig. 3). These genes were induced following *K. apiculata* application. A significant increase in the expression of the genes encoding for chalcone-flavanone isomerase (Cit.17011.1.S1_s_at), cinnamoyl-CoA reductase (Cit.13313.1.S1_s_at), violaxanthin de-epoxidase (Cit.30844.1.S1_s_at) and shikimate 5-dehydrogenase (Cit.25466.1.S1_at) was observed. These genes are mainly involved in to lignin and flavanol biosynthesis. Moreover, genes involved in the biosynthesis of the other secondary metabolites, such as carotenoid and terpenes, were down-regulated, including *p*-coumarate 3-hydroxylase (Cit.30567.1.S1_at), 3-chloroallyl aldehyde dehydrogenase (Cit.30574.1.S1_s_at), ent-kaurenoate oxidase (Cit.13587.1.S1_at) and carotenoid isomerase (Cit.29769.1.S1_s_at).

Several families of transcription factors, including the WRKY, R2R3-MYB, bHLH (basic helix-loop-helix) and WD40 genes, showed significant transcriptional changes in response to *K. apiculata* application, as revealed by the microarray data.

### Comparative analysis of gene expression in citrus between different treatment

To further analyze the response of the orange exocarp tissue to the extract and PEA, a total of 339 and 608 genes were identified. The expression profiles in response to the extract and PEA were similar to that found for *K. apiculata*; 57.4 % of the 803 DEGs in response to *K. apiculata* were also altered in the extract/PEA treatments (Fig. 1). The common up-regulated and down-regulated genes in citrus between *K. apiculata*-PEA and special in *K. apiculata* were listed in Additional file 4. The distribution of the genes among the GO and KEGG functional categories indicated that a large number of defence-related genes were also included in these DEGs. Orange exocarp tissue responded similarly to *K. apiculata*, the extract and PEA (Figs. 3 and 4).

### Verification of microarray data by qRT-PCR analyses

**Fig. 5:**
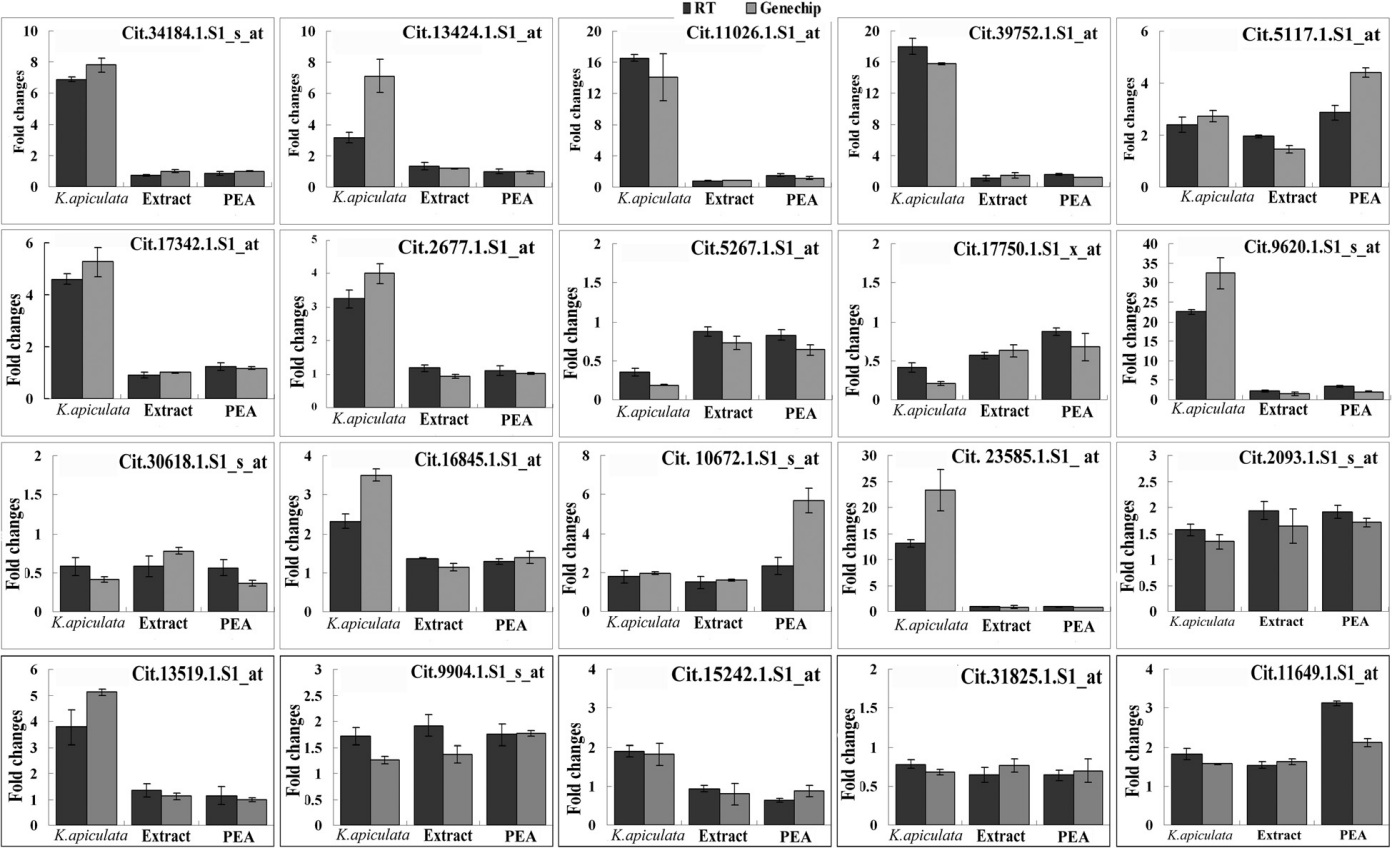
Verification of the microarray results by qRT-PCR. Black bar: qRT-PCR results for the genes. Grey bar: microarray data for the genes. Each qRT-PCR reaction was carried out in triplicate for three repeats. Columns and bars represent the means and standard error (*n* = 3) respectively

To confirm that the DEGs identified by the microarray gene expression were indeed differentially expressed, 20 genes were selected based on their biological significance for confirmation in a biologically independent experiment using qRT-PCR, including SOD-, ET-, JA- and ABA-related genes, which were detected in the microarray data and bioinformatic analyses. The relative transcript abundance patterns for the stress application were compared using the transcriptome data. The results of the qRT-PCR experiments revealed that most of the genes showed the same expression pattern as the microarray data (Fig. 5), such as Cit.2677.1.S1_at, which was 3.2-times higher in response to *K. apiculata*-treatment than in CK in qRT-PCR data, and 4.0-times higher for the microarray data.

### Pathogenesis-related (PR) proteins activity

**Fig. 6:**
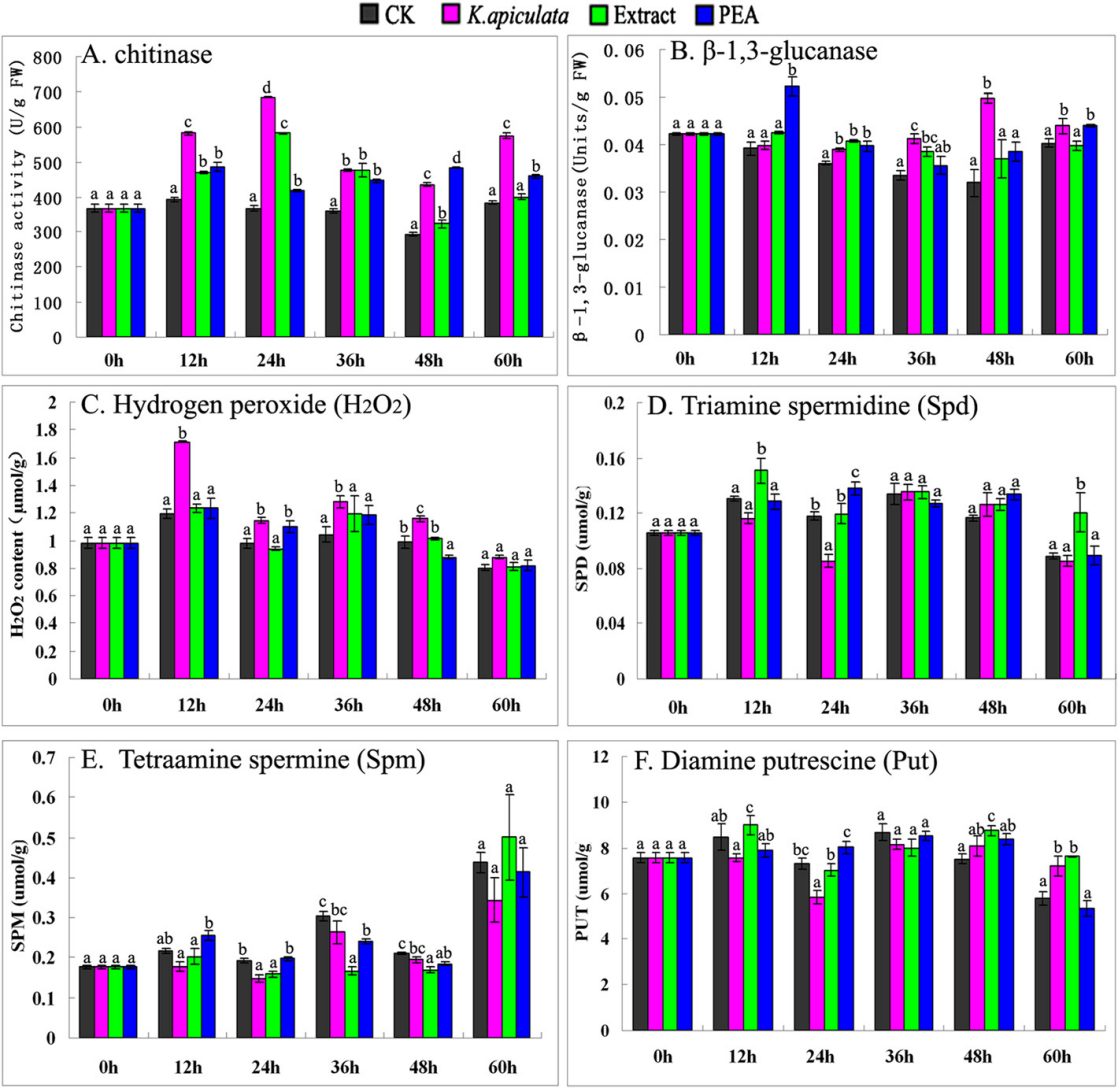
PR protein activity and polyamine and H_2_O_2_ content between *K. apiculata*, PEA and the extract treatment. **a** Chitinase activity; (**b**): β-1,3-glucanase activity; (**c**): H_2_O_2_ content; (**d**-**f**): polyamines content. The results in all the histograms are expressed as means ± standard errors. Mean values for different treatments at each time point are labelled with different letters to indicate significant differences at the level *P* < 0.05 according to Duncan’s multiple range test

**Fig. 7:**
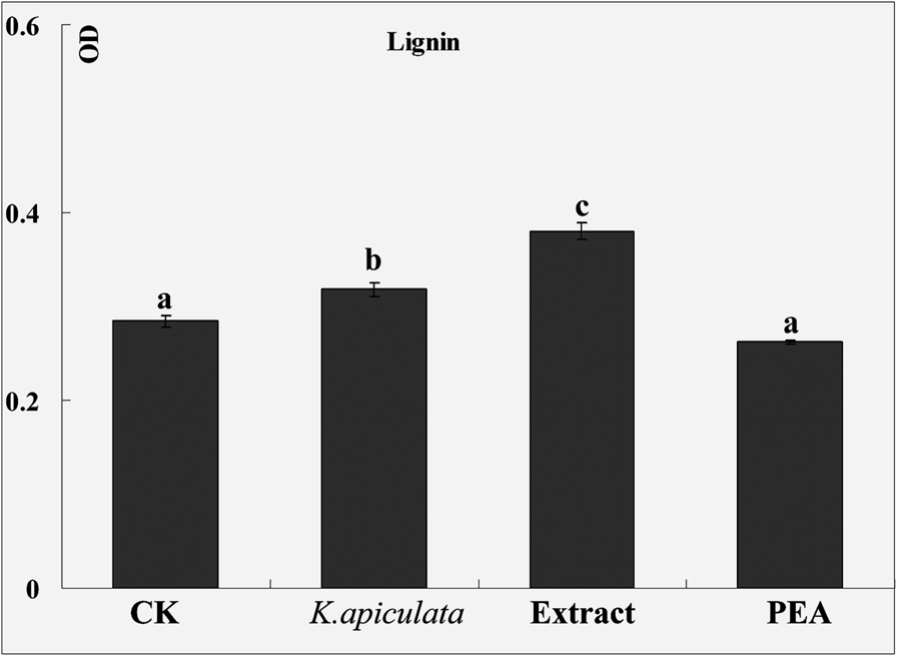
Lignin content between *K. apiculata*, PEA and the extract treatment. The results in all the histograms are expressed as means ± standard errors. Mean values for different treatments at each time point are labelled with different letters to indicate significant differences at the level *P* < 0.05 according to Duncan’s multiple range test

Of the PR proteins, chitinase and β-1,3-glucanase are two of the most fully characterized enzymes that are capable of hydrolyzing the polymers of fungal cell walls [27] Furthermore, the accumulation of chitinase and β-1,3-glucanase is important in retarding fungal growth and decreasing the spoilage of fruits caused by fungal pathogens. The level of chitinase and β-1,3-glucanase was observed to be significantly higher after inoculation of fruit with yeast, the extract and PEA compared with the control (Fig. 6ab), which is consistent with the microarray data. In addition, some PR-proteins could act in cell wall reinforcement by catalyzing lignification, such as PR-9 [46]. As illustrated in Fig. 7, the application of *K. apiculata* and the extract to discs of orange peel increased the lignin content in fruit peel relative to the water control peel discs 60 h after application.

### Hydrogen peroxide (H_2_O_2_) level in orange tissue

Reactive oxygen species (ROS) burst has been shown to regulate the yeast response processes [15]. Our study showed that *K. apiculata* treatment resulted in a high level of intracellular H_2_O_2_ when applied to oranges (Fig. 6c); this level decreased dramatically 12 h after the application of yeast to citrus fruit, although the statistic analysis showed that there were significant differences (*P* < 0.05) between *K. apiculata*-treatment and control at the point of 24 h, 36 h and 48 h. The extract and PEA did not enhance the level of H_2_O_2_.

### Polyamine level in orange tissue

Polyamines, mainly diamine putrescine (Put), triamine spermidine (Spd) and tetraamine spermine (Spm), act as an important source of H_2_O_2_ production and have been suggested to be involved in the response to pathogen attack or responsible for enhanced disease resistance in higher plants [47]. In yeast-treated citrus, the level of Put, Spd and Spm were observed to be lower than normal control, especially at 24 h (Fig. 6). In the extract and PEA-treated citrus, there was not a rule can be followed for Spm, Put and Spd (Fig. 6d, e, f).

## Discussion

Interactions between postharvest yeast biological control agents and host tissue have recently been widely studied in various fruits, including citrus, apples, pears; however, the molecular mechanisms are poorly understood. Gene expression profiling via the use of microarrays has been recognized as a powerful approach to obtain an overall view of gene expression and the physiological processes involved in the response to a particular stimulus [48–50]. In this study, we used a microarray to identify global changes in gene expression that occur in orange fruit exocarp tissues following the application of the yeast biological control agent *K. apiculata*, its extract and PEA. A large number of newly discovered and interesting genes encoding transcription and post-transcription factors were included in these DEGs, indicating that these genes may be key regulators that control the defence response by activating or repressing numerous genes. Additionally, a number of putative homologs of genes for host resistance were also found.

A variety of genes involved in the response to biotic and abiotic stresses, signalling, defence, hormones and secondary metabolism were identified in *K. apiculata*-treated orange exocarp tissue (Fig. 3). These findings imply that complex biochemical and molecular processes are involved in the reaction of fruit host tissue to the introduction of yeast cells, which have the potential to influence the efficacy of the biocontrol agent.

Plant ROS-signalling pathways have been shown to play essential roles in the regulation of host defence response processes [40, 51]. Previous data showed that the production of ROS by yeast antagonists may serve as a signal to trigger an oxidative burst in host tissue, leading to the activation of host defence mechanisms [17]. In contrast, fungal pathogens, such as *Penicillium expansum* and *P. digitatum*, suppress host cell defence responses by inhibiting the production of H_2_O_2_ in host cells [19, 20]. In the current study, a significant accumulation of H_2_O_2_ was observed in the host tissue after the application of *K. apiculata* to cells at 12 h (Fig. 6c). These finding were consistent with previous reports [17, 41]. The intensity of ROS production peaked shortly after the application of yeast cells to intact fruit, with a concomitant accumulation of H_2_O_2_ in the host fruit tissue itself [40]. Overall, these results support the notion that the intense production of ROS in fruit tissue induced by yeast cells plays a major role in the early stages of the application of *K. apiculata*.

Plant mitochondria have been reported as a source of the oxidative burst [52]. In our microarray data, 39 genes were finally categorized as mitochondrial by GO categories, including respiratory chain complex and electron transport. In addition, MapManBin identified three and six genes as being involved in mitochondrial electron transport/ATP synthesis and redox ((reduction-oxidation) reactions, respectively. The oxidative burst in fruit host tissue is likely responsible for the disordered of energy metabolism in mitochondria. ROS accumulation in yeast-treated tissue is also accompanied by a decrease in the expression levels of genes encoding for ROS-detoxifying enzymes, including monodehydroascorbate reductase, SOD, CAT and POD (Fig. 4). Changes in antioxidant gene expression, which lead to an increase in the ROS levels, and the activation of defence mechanisms have been supported by several reports [15, 18, 40, 51]. ROS contribute to the activation of plant defence by inducing changes in gene expression, including the redox regulation of transcription factors, production of PR proteins, ET synthesis and cell death [53].

Plant hormones play pivotal roles in the regulation of the defence signalling network [30]. The signalling pathways crosstalk in an antagonistic or synergistic manner, providing the plant with a powerful capacity to finely regulate its immune response [54]. SA, Jas and ET are recognized as major defence hormones [55]. Other hormones, including ABA, auxin and gibberellins, affect the SA-JA-ET backbone of the plants immune signalling network, resulting in positive or negative effects on biotrophic and necrotrophic pathogens [56–58]. In our microarray data, we detected a significant increase in JA-signalling (8 genes) and ET-signalling (6 genes) gene expression in yeast-treated orange exocarp tissue. This finding was further supported by qRT-PCR data (Fig. 5), and the decreased Spd and Put levels (Fig. 6df) also supported these results. ET and polyamines have a common precursor, and they appear to have opposing physiological roles [59]. In addition, our results identified a difference in the expression of 27 genes related to other hormones (polyamine, gibberellins, auxins and ABA). Their signalling pathways may have indirect effects on plant immunity by antagonistically or synergistically interacting with the SA-JA-ET backbone of the plant immune signalling network [55]. The data supported the *K. apiculata*-induced citrus defence response via the JA/ET-signalling pathway.

Following *K. apiculata* treatment, 57.4 % of DEGs showed the same pattern of change as was found following the extract/PEA treatments (Fig. 1 and Additional file 4). This result indicated that the extract and PEA have the potential to influence the efficacy of the biocontrol agent of *K. apiculata* by inducing defence response genes. Furthermore, the ability of *K. apiculata* to induce the defence response of citrus is partly related to the extract and PEA. The global expression profiles were similar in response to treatment with *K. apiculata*, the expression of defence-associated genes being greatly enhanced under the extract- and PEA-treatment (Figs. 3 and 4). Meanwhile, we noticed that application of the yeast strain enhanced larger effects than application of the extract or PEA, which may be interpreted the effects of *K. apiculata* involved in both biotic and abiotic stress. Only abiotic stress of *K. apiculata* was partly achieved via secretion secondary metabolite, such as PEA.

As a metabolite of L-Phe, PEA may negatively regulate the biosynthetic pathway and indirectly influence the production of a fruit’s L-Phe-derived metabolites [60]. These metabolites have protective and regulatory functions in plants and can be categorized into three broad groups: phytoalexins (flavonoids, isoflavanones), phytoanticipins and signalling molecules (e.g., SA). Flavonoid glycosides serve as potential modulators of cell division, while flavonoids serve as regulators of auxin transport and SA acts as a regulator of both local and systemic pathogen-induced defence gene activation, the oxidative burst and pathogen-induced cell death [56, 61]. All L-Phe-related genes in the *K. apiculata* and PEA treatments (Cit.16303.1.S1_at, Cit.1280.1.S1_s_at, Cit.29769.1.S1_s_at, Cit.6742.1.S1_s_at, Cit.9171.1.S1_at, Cit.9944.1.S1_at, Cit.9944.1.S1_x_at, Cit.17011.1.S1_s_at, Cit.12979.1.S1_at) shared the same up- and down-regulated pattern as the control, except for Cit.15355.1.S1_at.

## Conclusion

**Fig. 8:**
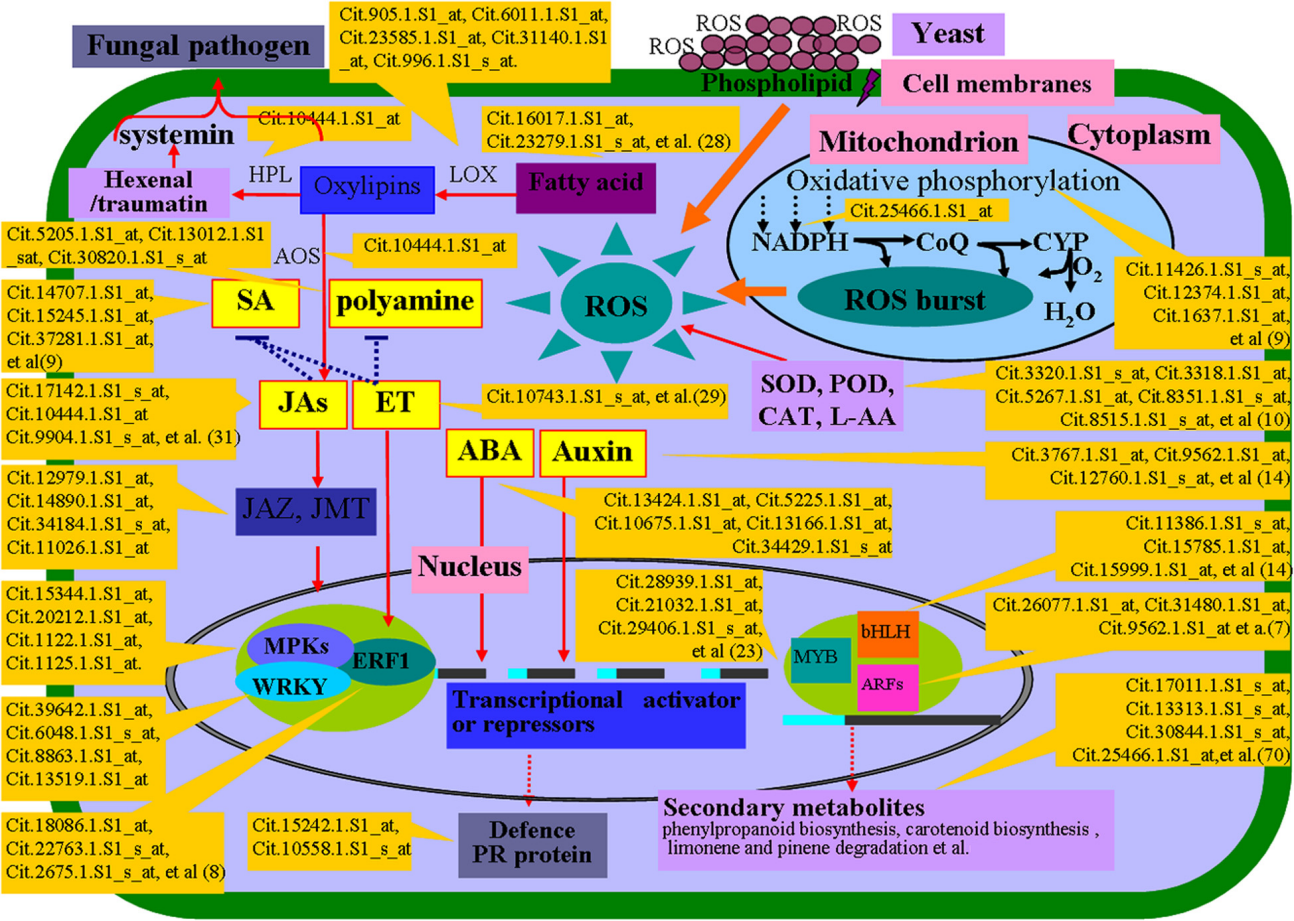
Overview of the major metabolic pathways involved in the defence response, as suggested by the interpretation of the GO and KEGG. The genes are designated as ID number from Citrus GeneChip, and the detailed gene information can be viewed in Additional file 1: Table S1. JA: jasmonic acid, ABA: abscisic acid, ET: ethylene, ROS: reactive oxygen species, LOX: lipoxygenase, HPL: hydroperoxide lyase, L-AA: L-ascorbic acid

Our study provides a global picture of the gene expression changes that result from the application of the yeast biological control agent *K. apiculata*, its extract and PEA on citrus fruit (Fig. 8). The microarray data revealed a large number of genes that were reported to be involved in the defence response. The functional categorization of the DEGs revealed the involvement of a number of important pathways, including oxidative phosphorylation, phenylpropanoid biosynthesis, mitochondrial electron transport/ATP synthesis, MAPK signalling, calcium signalling and hormone cross communication, in regulating of the defence process. In addition, similar global expression profiles were acquired with the expression of defence-associated genes between *K. apiculata*, the extract and PEA treatments.

## Methods

### Fruit material and biocontrol agent

Olinda Valencia oranges (*Citrus sinensis* L. Osbeck) were harvested at commercial maturity (28 April) from adult trees grown in Yichang City, Hubei Province, China. Fruits without physical injuries and infections were selected based on uniformity in size. Prior to use, the fruits were disinfected with 2 % (*v/v*) NaOCl solution for 2 min, rinsed with tap water and air-dried. A strain of *K. apiculata* 34–9 was isolated from the epiphytes of citrus roots [5]. The strain was grown in YPD medium (1 % yeast extract, 2 % peptone, 2 % dextrose and 2 % agar).

### Citrus RNA extraction and microarray analysis

The extract was obtained from the cell-free culture of *K. apiculata* as described previously [42]. Citrus fruits soaked for 5 min in 1.0 × 10^8^ cells/mL *K. apiculata* (KA), 1530 μg/mL PEA, the extract (1000 × dilute) and water control (CK), then air dried and the samples were then placed on plastic cases for 24 h. The fresh exocarp of citrus was separated with a knife after washing the fruit with water, after which it was directly frozen in liquid nitrogen and stored at −80 °C. Each sample consisted of the pooled exocarp of six fruits. Two biological replicates were used for each line.

The total RNA was extracted as described previously [62]. The Affymetrix GeneChip One-cycle Target Labeling Kit (Affymetrix, Santa Clara, CA; http://www.affymetrix.com/) was used for expression analysis. The GeneChip Citrus Genome Array (platform: GPL5731) contains 30,171 probe sets representing up to 33,879 citrus transcripts based on EST sequences obtained from several citrus species and citrus hybrids. The arrays were performed according to the manufacturer’s recommended protocols. Microarray experiments were designed to comply with MIAME guidelines [63]. The differentially expressed genes (DEGs) were selected and functionally annotated as described in Gallego-Giraldo et al. [64]. We used the classical ttest to identify DEGs and defined *p*-value < 0.05 to be statistically significant. The details of the citrus cDNA microarray data were submitted to NCBI under GEO accession numbers GSE45680.

### Quantitative real-time RT-PCR (qRT-PCR)

The total RNA was reverse transcribed into first-strand cDNA using the M-MLV first strand kit (Invitrogen, USA) according to the manufacturer’s instructions. Twenty genes were chosen for confirmation by qRT-PCR with SYBR®GREEN Master Mix (Toyobo, Osaka, Japan). Primers for the chosen genes were designed with the primer express software (Applied Biosystems, USA) and are presented in Table 1. A qRT-PCR assay for gene expression analysis was performed on a Roche 480 Real-time PCR System (Roche Molecular Systems, Belleville, USA) using the beta-actin (ACTB) gene as an endogenous control according to Yan et al. [65]. Briefly, the primers for the target gene and ACTB were diluted in the SYBR Mix, and 10 μL of the reaction mix were added to each well. The reactions were performed with an initial incubation at 50 °C for 2 min and at 95 °C for 1 min followed by 40 cycles of 95 °C for 15 s, 60 °C for 20 s and 72 °C 10 s. The levels of gene expression were analysed with a LightCycler®480. Zero template controls were included for each primer pair. Each PCR reaction was carried out in triplicate for three repeats, and the data were presented as the means ± SD.

**Table 1:** Sequences of primers used in real-time PCR

Gene name	Accession number	Primers(5′-3′) Forward	Reverse
JAZ10	Cit.34184.1.S1_s_at	TTAAATCTGTGATTGCTTCTGGTTCT	GGGAAAAGTAATCGGCTCTTCTT
CYP707A3	Cit.13424.1.S1_at	GGGCACAAATACTGGAATCCA	TCTGATGGGCAATCCATTCA
JAZ10	Cit.11026.1.S1_at	GTAGTTTTGTAGAAATTCTGTTTCCATCTT	CAAACGAGGCAGGCTATTAAGG
ATEXKA2	Cit.39752.1.S1_at	TGTGTGCACCAAACCAAGGT	CACATGCCCCGGATGAAA
USP	Cit.5117.1.S1_at	CATCAAGGCCGTCGAGAAAT	ACAGCCTCAGCTCGATTCATC
ATCM2	Cit.17342.1.S1_at	TGCCCTTTACCAAGCTCGTT	AGCTAGCACAGGTTTCAACTTCAA
AP2-EREBP	Cit.2677.1.S1_at	CCCTTTGACGCCGTCAAG	GCGGCACACTAAATATCCCATT
SOD	Cit.5267.1.S1_at	CTGTTGATGTTGACGGTACTGCTA	CCAATAACAGAATCTGGTCCAGAA
Unknown	Cit.17750.1.S1_x_at	CTCCAGAAGCTAACAGAGATCGTTT	CCCTCTGACCATTCTTTGTTACCT
Unknown	Cit.9620.1.S1_s_at	TGCTTGCAGCCAGATAAGTGTAC	CAACAAAGGCATTAACCCACAA
MYB	Cit.30618.1.S1_s_at	CCTCCTCGACCAAAACGAAA	AACAGGTGGATGGGCCAAT
ERF12	Cit.16845.1.S1_at	CGTCCTTCTTTGGGATTGTGA	GCGGTGGCGGCTCAT
CCL	Cit. 10672.1.S1_s_at	TGCTCGGCAGAGATGTATCCT	AGGCAGTTGTTGATGTGTCGTT
LEA14	Cit. 23585.1.S1_ at	AAGAGGGCGGATTTAGTTTTTAGAT	AATGTTGCTTTCTTTACGTGCATAA
EXPA5	Cit.2093.1.S1_s_at	CTGTATCCATCAAAGGTTC	GGTATAAGTCTGCCCAAA
WRKY40	Cit.13519.1.S1_at	CCCGTAAAGAAGAAGGTG	GCCGCAGAAACATGAATA
LOX2	Cit.9904.1.S1_s_at	GTTTGGGTAACATCTGGT	CTTGAATCTGGGAAGGGA
Chitinase	Cit.15242.1.S1_at	CTGCCTTGTGGTTTTGGA	AACTTTATCGGGCTGCTT
PR protein	Cit.31825.1.S1_at	GACTTGTAACTATGACCCTG	ACTTGCTACTGTCGCTAA
Esterase	Cit.11649.1.S1_at	TTTCCCTGGACTTTTCTAC	ATCAAATTCCCATGTGCC
ACTB	Reference	CCAATTCTCTCTTGAACCTGTCCTT	GAAGACCGTCAAGAGTAGTCAGT

### Plant endogenous H_2_O_2_, lignin and enzyme activity analyses

The disinfected fruits were inoculated on their circumference using an inoculating needle (5.0 mm). For inoculations, a 10 μL aliquot of the yeast suspension at a concentration of 1.0 × 10^8^ cells/mL was dropped onto each prick. After air drying, the fruits were stored in enclosed plastic trays to maintain a high humidity (approximately 95 %). The plastic cases were maintained at 28 °C for the indicated periods. To measure the elicitation effect, the tissue surrounding each wound of fruit was collected at hour 0, 12, 24, 36, 48, 60 after treatment, and immediately immersed in liquid nitrogen and stored at −80 °C until use. A 10-g sample (fresh weight; FW) of the exocarp was ground into a powder in liquid nitrogen.

The concentration of H_2_O_2_ was assayed using H_2_O_2_ assay kits (Nanjing Jiancheng Bioengineering Institute, China) according to the manufacturer’s instructions. The enzyme activities were determined by a Shimadzu UV-1800 spectrophotometer (Shimadzu, Japan). The activities of chitinase and β-1,3-glucanase were measured as described previously [11]. The lignin content was quantified using the method described by Syros et al. [66].

### Quantification of free polyamines by high-performance liquid chromatography (HPLC)

The free polyamines were quantified using a method previously described in Liu and Moriguchi [67] and Fu et al. [47]. Samples were prepared and collected as described above. A sample of peel tissues (0.5 g) was homogenized in 5 mL of 5 % cold perchloric acid (PCA) for 30 min on ice. The supernatant was transferred to a new tube after centrifugation at 12000 rpm (4 °C) for 15 min; the resulting pellet was reconstituted with 5 mL of 5 % PCA and maintained on ice for 30 min before centrifugation at the same conditions. The supernatant was mixed, and 500 μL of it was benzoylated. The supernatant was mixed with 10 mL of benzoyl chloride and 1 mL of 2 mol NaOH. The resultant solution was vortexed for 30 s and then incubated for 25 min in a water bath at 37 °C. The benzoylated polyamines were then leached with 2 mL of ethyl ether, vacuum dried in a concentrator (Eppendorf 5301, Germany) and re-dissolved with 100 μL of methanol (HPLC grade). The benzoyl-polyamines (20 μL) were analysed using an Agilent 1200 HPLC systems (Santa Clara, CA, USA) equipped with a C_18_ reversed phase column (4.6 mm × 150 mm, particle size 5 μm) and a UV-detector according to Shi et al. [68] with minor modification. The column was eluted at 1 mL/min, with a programmed gradient of solvents (methanol/water), changing from 60 to 95 % in 23 min. Chromatograms were scanned at 230 nm. The polyamines were quantified in triplicate.

### Statistical analysis

All the statistical analyses in this study were conducted using the Statistical Program SPSS 13.0 for windows (SPSS Inc, Chicago, IL). Analysis of variance (ANOVA) was performed and Duncan’s multiple range test was used for means separation. The statistical significance in this experiment is all applied at the level *P* < 0.05.

## Additional files

Additional file 1: Table S1. List of differentially expressed genes and GO and KEGG analyses between *K. apiculata*, the extract and phenylethanol treatment. The table contained information of the differential expressed genes with expression difference, and genes differential expressed at 0.05 significance level. (XLS 1869 kb) Additional file 2: Table S2. List and MapMan analysis of differentially expressed genes in citrus in response to *K. apiculata*, the extract and phenylethanol, respectively. (XLS 264 kb) Additional file 3: Table S3. List of defence-related differentially expressed genes in citrus under *K. apiculata* treatment. (XLS 115 kb) Additional file 4: Table S4. The common or in special up-regulated or down-regulated expressed genes in citrus between *K. apiculata*, PEA and the extract treatment. (XLS 301 kb)
